# A nucleolar localizing Rev binding element inhibits HIV replication

**DOI:** 10.1186/1742-6405-3-13

**Published:** 2006-05-19

**Authors:** Alessandro Michienzi, Fernanda G De Angelis, Irene Bozzoni, John J Rossi

**Affiliations:** 1 Division of Molecular Biology, Beckman Research Institute of the City of Hope, 1450 East Duarte Rd., Duarte, California 91010, USA; 2 Istituto Pasteur, Fondazione Cenci-Bolognetti, Department of Genetics and Molecular Biology, University of Rome La Sapienza and IBPM of CNR, Rome, Italy; 3 Present address: Istituto Superiore di Sanita', Department of Cell Biology and Neuroscience, Viale Regina Elena 299, 00161, Rome, Italy; 4 Division of Molecular Biology, Graduate School of Biological Sciences, Beckman Research Institute of the City of Hope, 1450 E. Duarte Rd., Duarte, CA 91010, USA

## Abstract

The Rev protein of the human immunodeficiency virus (HIV) facilitates the nuclear export of intron containing viral mRNAs allowing formation of infectious virions. Rev traffics through the nucleolus and shuttles between the nucleus and cytoplasm. Rev multimerization and interaction with the export protein CRM1 takes place in the nucleolus. To test the importance of Rev nucleolar trafficking in the HIV-1 replication cycle, we created a nucleolar localizing Rev Response Element (RRE) decoy and tested this for its anti-HIV activity. The RRE decoy provided marked inhibition of HIV-1 replication in both the CEM T-cell line and in primary CD34+ derived monocytes. These results demonstrate that titration of Rev in the nucleolus impairs HIV-1 replication and supports a functional role for Rev trafficking in this sub-cellular compartment.

## Background

HIV-1 gene expression is finely regulated [1]. Transcription from the HIV-1 long terminal repeat (LTR) produces a full length RNA of 9 Kb from which several mRNAs are then generated by splicing (the 4 Kb singly spliced and 2 Kb fully spliced RNAs) [1]. While the fully spliced RNAs are exported to the cytoplasm, where they are translated into the regulatory and accessory proteins, the late structural proteins and reverse transcriptase (RT) are derived from unspliced or singly spliced RNAs which are transported to the cytoplasm only upon binding of Rev to the Rev Responsive Element (RRE), which is contained in the *env *coding region [1]. Rev is an 18kDa protein that localizes to both the nucleus and nucleolus [2-4]. It contains a nuclear export signal (NES) as well as a nuclear import signal (NLS) that allow nuclear/nucleolus-cytoplasmic shuttling properties [5,6]. The NES signal of Rev is recognized by the cellular export factor CRM1, which mediates the nuclear-cytoplasmic export of Rev bound RNAs [7,8]. Expression of Rev in human cells induces re-localization of CRM1 and some nucleoporins (Nup98 and Nup214) into the nucleolus [9]. Recently, by the use of *in vivo *fluorescence resonance energy transfer (FRET), multimerization of Rev-GFP and BFP fusion proteins has been shown to occur in the nucleoli of HeLa cells [10]. These observations suggest that the nucleolar trafficking of Rev may be critical for Rev mediated export. To test this hypothesis we used a nucleolar localized decoy that contains the Rev binding element (RBE) [11-14] to sequester Rev within this sub-cellular compartment and test its ability to inhibit HIV-1 replication. The well-characterized U16 small nucleolar RNA (snoRNA) [15] was used to direct nucleolar delivery of the RBE.

In the present study we demonstrate that stable expression of the U16-RBE chimeric RNA in cultured T-cells and primary monocytes confers strong inhibition of viral replication. These data provide strong evidence that the nucleolar localization of Rev is critical for its functional role in HIV-1 replication and identifies a novel mechanism for inhibition of HIV replication.

## Materials and methods

### Plasmid constructs

**Figure 1:**
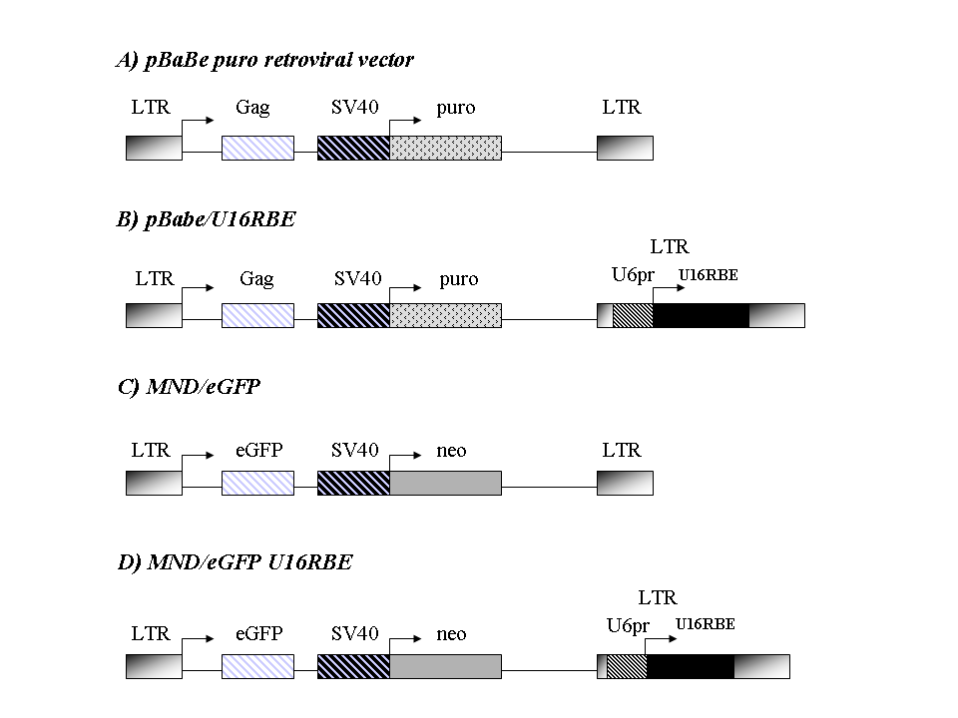
**Retroviral constructs**. (A) Schematic representation of the pBabe puro retroviral vector [22]. (B) Schematic representation of the pBabe/U16RBE construct. The PTZU16-RBE cassette was inserted in the U3 region of the 3'LTR, with the U6 promoter. Transcription is oriented in the same direction as the RNA pol II LTR and SV40 promoters. (C) Schematic representation of the MND/eGFP retroviral vector. (D) Schematic representation of the MND/eGFP U16RBE construct. The PTZU16-RBE cassette was inserted in the U3 region of the 3'LTR, with the U6 promoter transcribing in the same orientation as the RNA pol II LTR and SV40 promoters.

The U16RBE DNA was prepared synthetically as previously described [14]. The Rev Binding Element (RBE) was inserted in the U16 snoRNA sequence by replacing the apical loop [14]. The U16RBE sequence was then subcloned within the *Sal*I and *Xba*I sites of the pTZU6+1 expression cassette [16] generating the pTZU16-RBE clone. The *Bam*HI and *Xba*I cleaved fragment from the pTZU16-RBE construct was first filled in with the DNA polymerase Klenow fragment and then inserted in the *Nhe*I site of the pBabe puro retroviral vector (in the U3 region of the 3'LTR, Fig. 1A) giving rise to the pBabe/U16RBE clone (Fig. 1B). The U6 promoter-U16RBE was also cloned into a *Stu*I site in the U3 region of the MND/eGFP Banshee retroviral vector 3'LTR (Fig. 1C), generating the MND/eGFP U16RBE construct (Fig. 1D).

**Figure 2:**
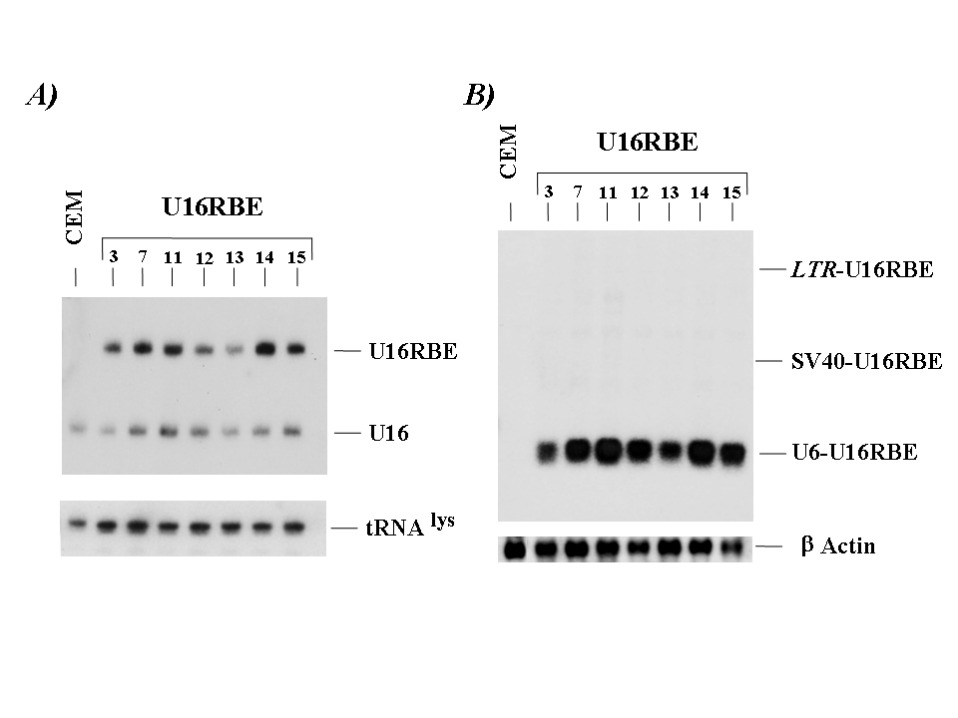
**Stably transduced CEM clones expressing the U16RBEdecoy**. The CEM cells were first transduced with the pBabe constructs and then selected for Puromycin resistance. From the resulting pooled population single clones were selected by limiting dilution. Total RNA isolated from the single clones was electrophoresed either in a 6% polyacrylamide-7M Urea (A) or 1.2 % Agarose formaldehyde (B) gels.  The electrophoresed RNAs were blotted onto a nylon filter followed by hybridization with specific probes (see Material and Methods).

### Cell culture

HEK 293, CEM and the PG13 packaging cells were maintained as previously described [17]. Transient transfections were carried out using a Calcium Phosphate DNA precipitation kit in accordance with the manufacturer's protocol (GIBCO/BRL, Invitrogen). Briefly, 1 × 10^6 ^HEK 293 cells were plated one day prior to transfection. The transfection was carried out using 2–10 μg of plasmid DNA.

### Packaging cell line

PG13 was used for packaging of the pBabe/U16RBE and MND/EGFP U16RBE constructs [17].

### CD34+ cell isolation and transduction procedures

CD34+ cells were isolated from fresh umbilical cord blood using anti-CD34+ antibody-coupled magnetic beads (Milteny Biotech, Auburn, CA). Sorted CD34+ cells (purity was above 90% as determined by FACS analysis) were cryopreserved until use for transductions by the viral vectors.

One day prior to transduction, CD34+ cells were thawed rapidly at 37°C, washed and plated at a concentration of 2 × 10^5 ^onto Retronectin (Takara, Japan) coated 25 cm^2 ^flasks. The Retronectin concentration for coating was 20 μg/cm^2^. The CD34+ cells were then pre-stimulated in 5 ml of cytokine media: Iscove's Modified Dulbecco's Media (IMDM – Bio Whittaker, Walkersville, MD), with 20% FBS, 100 U/ml Penicillin, 0.1 mg/ml Streptomycin (Sigma, St. Louis), 2 mM Glutamine (Sigma, St. Louis), IL-3 (10 ng/ml), IL-6 (10 ng/ml), and SCF (10 ng/ml) (R&D Systems, Minneapolis, Minnesota). After 24 hours of pre-stimulation, the media was changed and the CD34+ cells were exposed to the retroviral vector supernatants (MND/eGFP, MND/eGFP U16RBE and Mock). 50% percent retroviral vector supernatant and 50% 2 × cytokine media were used in a total volume of 10 ml per 25 cm^2 ^flask. The 2 × cytokine media has the same composition as the cytokine media but the growth factor concentrations are doubled (20 ng/ml for all cytokines). After 24 hours of infection, the cells were centrifuged, the media removed, and the cells exposed to fresh retroviral supernatant and cytokine media in a total volume of 10 ml.

This process was repeated one more time, leading to 3 rounds of transduction.

After 3 rounds of transduction, CD34+ cells were immediately plated into a Colony Forming Units assay (CFU-assay) or Long Term Bone Marrow Cultures (LTBMCs).

### Colony Forming Unit assays

1 × 10^3 ^and 2 × 10^3 ^transduced CD34+ cells from each vector arm were plated into 1 ml semi-solid methylcellulose media (Methocult, Stem Cell Technologies, Vancouver, Canada). After 14 days, colonies were counted and discriminated into Burst Forming Units Erythroid (BFU-E), Granulocyte-Macrophage Colony Forming Units (CFU-GM) and Granulocyte- Erythroid- Macrophage- Megakaryocyte (CFU-GEMM) Colony Forming Units. The colonies for each vector arm were added and the data expressed as a total colony count per group.

### Long term bone marrow cultures

Transduced CD34+ cells were plated into 25 cm^2 ^flasks onto a layer of irradiated normal human bone marrow stroma in cytokine media. The cytokine concentrations for SCF, IL-3 and IL-6 were 10 ng/ml each.

Normal human bone marrow was purchased from Bio Whittaker (Cambrex Bio Science Inc. Rockland, MD), 5 ml were plated directly into Dulbecco's Modified Eagle Media (Cambrex Bio Science) with 10% Fetal Bovine Serum (GIBCO) in a 75 cm^2 ^flask. Bone marrow cells were allowed to adhere overnight. Media and floating cells were removed, and fresh media were added. Stromal cells were allowed to expand for about 4 weeks. After expansion, stromal cells were trypsinised and cryopreserved for later use, or irradiated (2000 rads) and plated into 25 cm^2 ^flasks for LTBMCs at a density of 1 × 10^5 ^cells per flask.

CD34+ cells were allowed to expand in the stromal culture flasks. Cell samples were taken for eGFP FACS analysis 2 days after plating to assess the transduction frequency.

10 days after plating, cultures were depleted of the floating cell population, which was used for eGFP cell sorting and subsequent HIV-challenge. After depletion, the remaining cell fraction in the stroma flasks regenerated a high number of floating cells within 14 days. After 6 weeks of LTBMC propagation, a final FACS analysis was performed to assess eGFP expression.

### eGFP sorting and propagation of sorted CD34+ progenitor derived cell populations

CD34+ derived cells were sorted for eGFP expression in a MoFLO (Cytomation) cell sorter into 6 well plates containing a layer of irradiated normal human stroma and 2 × cytokine media (20 ng/ml all cytokines). Cells were expanded in these 6 well plates for approximately 5 days. After expansion, cells were removed from the wells and divided into 2 fractions: 1 × 10^5 ^cells for eGFP FACS analysis (purity check), and 1 × 10^6 ^cells for HIV-challenge.

### HIV-challenge

CEM cells (2.5 × 10^5^) derived from stably transduced clones were infected with HIV-1_NL4-3 _as previously described [18]. For CD34+ derived monocytes, 1 × 10^6 ^sorted CD34+ cells were transferred into 12 × 75 tubes, centrifuged, and resuspended in 100 μl of 2 × cytokine media. 100 μl of HIV-1 JRFL with a titer of 1 × 10^5 ^/ml was added to each challenge tube, resulting in a multiplicity of infection (m.o.i.) of 0.01. Cells were incubated at 37°C and 5% CO_2 _for 3 hours and then 200 μl of 2 × cytokine media were added to each tube and the cells were incubated overnight.

On the following day, cells were washed 4 times and plated into 25 cm^2 ^flasks onto a layer of irradiated S17 cells (kindly provided by Kenneth Dorshkind, UCLA), in 1 × cytokine media in a total volume 10 ml. The cultures were propagated for 28 days and samples were taken weekly. Infections were all performed in duplicate or triplicate.

### HIV-1 p24 evaluation

**Figure 3:**
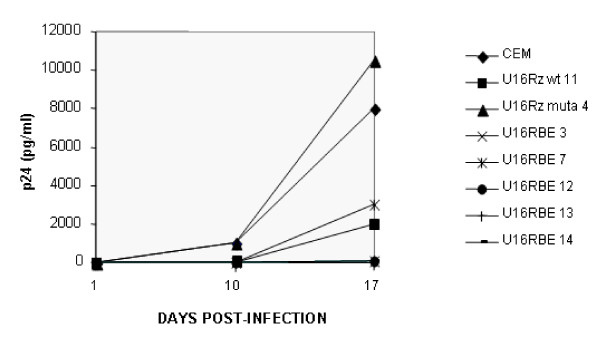
**U16RBE *in vivo *activity**. Some of the CEM clones expressing the U16RBE RNA decoy along with the parental CEM cells were challenged with HIV-1 NL4-3 at an m.o.i of 0.001.CEM clones expressing either a nucleolar anti-HIV-1 hammerhead ribozyme wt (U16Rz wt 11) or the mutant disabled mutant version (U16Rz mutant 4) were used respectively as positive and negative controls for inhibition of HIV-1 infection [18]. On days 1, 10 and 17 day post-infection the supernatants from these cell cultures were collected and assayed for HIV-1 p24^Gag ^accumulation. The data presented represent an average of two independent challenge experiments, and the standard errors for each point are around 5%

**Figure 4:**
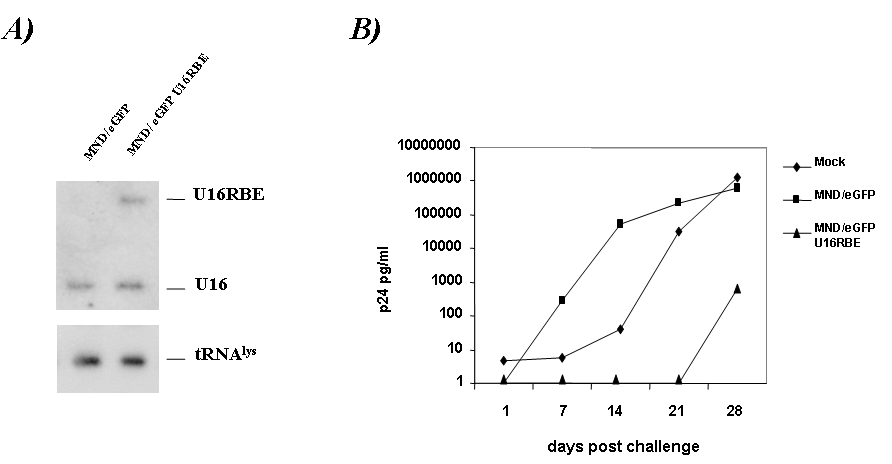
**CD34+ progenitor cell derived monocytes stably expressing U16RBE are resistant to HIV-1 infection**. CD34+ cells isolated from human cord blood were first transduced with either the parental MND/eGFP vector or the MND/eGFP U16RBE and then sorted for eGFP expression. (A) total RNA isolated from the eGFP sorted, transduced CD34+ cells was electrophoresed in a 6% polyacrylamide-7M Urea gel and blotted onto a nylon filter. Hybridizations were performed using a probe complementary to the 3' end of the U16 snoRNA and a probe specific for the tRNA_3_^Lys ^used as a loading control. (B) Parental CD34+ cells along with the transduced and eGFP sorted CD34+ cells were challenged with HIV-1 JFRL using an m.o.i. of 0.01. On days 1, 7, 14, 21 and 28 days post-infection the accumulation of the viral p24^gag ^antigen was assayed. The data presented represent an average of two independent challenge experiments, and the standard errors for each point are approximately 5%

The p24 analyses were performed using the HIV-1 p24 antigen capture assay kit (Science Applications International Corp. Frederick) for the experiment in Fig. 3 and a Beckman/Coulter p24 assay kit (Beckman, FL) for the experiments in Fig. 4B.

### RNA extraction and Northern Blot analysis

Total RNA was isolated as previously described [18]. Northern Blot analyses were performed by electrophoresing the isolated total RNA either in a 6% polyacrylamide-7M Urea gel or in a 1. % Agarose formaldehyde gel followed by blotting onto a nylon filter. To simultaneously detect the endogenous U16 snoRNA and the U16RBE RNA, a probe complementary to the 3' end of the U16 was used (Fig. 2A). To detect only the U16RBE a probe complementary to the RBE sequence was used (Fig. 2B). As loading controls probes specific for tRNA_3 _^Lys ^or the β-Actin were used (Fig. 2).

## Results

Nucleolar expression of the Rev Binding Element (RBE) was achieved by substitution of the apical loop of the U16 small nucleolar RNA (snoRNA) with the RBE sequence as previously described [14]. The RBE domain is located within the Rev responsive element (RRE) sequence (within stem-loop IIB, which contains a purine rich "bubble"). Rev has a high binding affinity for this element [11-13]. U16 is a member of the C/D box class of snoRNAs that are primarily involved in post-transcriptional modifications (2' O-methylation) of rRNA molecules [19]. In order to achieve expression of U16RBE in human cells, the chimeric gene was cloned downstream the U6 small nuclear RNA Pol III promoter (clone PTZU16-RBE; [14]). Intracellular expression and nucleolar compartmentalization of the U16RBE RNA in 293 cells has been previously demonstrated [14].

To test the intracellular efficacy of the U16RBE against HIV-1 replication, the PTZU16-RBE expression cassette was inserted into the U3 region of the pBabe puro retroviral vector (clone pBabe/U16RBE, Fig. 1). The resulting vector pBabe/U16RBE was then used to transduce the human CEM T cell line. The U6-U16RBE gene inserted within the U3 region of the retroviral vector should be duplicated within the 5' LTR following reverse transcription and integration. Single clones stably expressing U16RBE were isolated following Puromycin selection. Northern blot analyses performed using total RNA extracted from the selected clones showed somewhat varying levels of expression of the RNA decoy (Fig. 2A).

Transcription of the U16RBE could originate from the U6 promoter as well as from the 5' LTR and internal SV40 polII promoters (Fig. 1B). In order to discern the origins of the major transcripts, a Northern blot analysis was performed on RNA separated in a 1.2% Agarose-Formaldeheyde gel to resolve both long and short transcripts. As shown in Fig. 2B, more than 95% of the U16RBE RNA was of a size corresponding to a U6 promoter-driven transcript. Upon longer exposure (data not presented) transcripts of a size originating from the LTR and SV40 promoters can be seen. These results suggest that the majority of the U16RBE RNAs were derived from the Pol III promoters, with minor amounts originating from the LTR or SV40 internal promoters. In addition, transcripts originating from the Pol II promoters that read through the U16RBE structure may be subject to processing by the snoRNA processing machinery. It is not possible to determine how much, if any processing such as this takes place.

We next tested the anti-HIV-1 activity of the U16RBE decoy by challenging several of the stably transduced CEM clones expressing the U16RBE RNA with the HIV-1 _NL4-3 _strain using a multiplicity of infection of 0.001. As positive and negative controls for this experiment we also challenged CEM clones expressing either a nucleolar localizing anti-HIV-1 wild type ribozyme (wt) or its disabled mutant version (clones U16Rz wt and mutant; [18]).

At days 1, 10 and 17 post-infection the cell culture supernatants were collected and assayed for the release of the viral p24^Gag ^protein. As shown in Fig. 3, the controls (parental CEM cells and a CEM clonal line expressing the mutant U16Rz) were highly permissive to replication during the 17 days of analysis. In contrast, all the clonal lines expressing U16RBE and the one expressing U16Rz wt were highly resistant to the HIV-1 challenge.

To test the anti-HIV-1 activity of U16RBE in more clinically relevant cells, the PTZU16-RBE cassette was inserted within the U3 region of the 3'LTR of the MND/eGFP retroviral vector (Fig. 1C). This vector allows use of eGFP expression to monitor transduction. The recombinant vector was transduced into human CD34+ progenitor cells purified from umbilical cord blood. Cells were propagated under cell culture conditions that allow differentiation into CD4+ monocytes. Cell counts after pre-stimulation and transduction were similar for both mock and recombinant vectors, with cell viability greater than 90% and cell expansion about two-fold in all cases (data not presented). To assess potential toxicities of the vectors, colony assays were carried out using the transduced CD34+ cells. Although some differences in colony forming numbers were observed between the three samples (Table 1A), in our hands these small differences are not significant, suggesting no overt toxicity of the U16RBE expression.

**Table 1:** CD34+ cell transduction with MND constructs. (A). 1 × 10^3 ^and 2 × 10 ^3 ^transduced CD34+ cells transduced with the control or U16RBE expression vectors were plated into 1 ml semi-solid methylcelluose media. After 14 days, colonies were counted and analyzed for Erythroid-Burst Forming Units (BFU-E), Granulocyte Macrophage -Colony Forming Units (CFU-GM) and Granulocyte Erythroid Macrophage Megakaryocyte Colony Forming Units (CFU-GEMM). (B) After transduction CD34+ cells were allowed to expand in stroma flasks, and cell samples were taken for eGFP FACS analysis 1 and 45 days after plating to assess transduction frequency. The loss of eGFP expression with time is a consequence of epigenetic silencing of the MND LTR (Castanotto, Li and Rossi, unpublished results).

	**(A) CD34+ CFU Count Total**		
	BFU-E	CFU-GM	CFU-GEMM
MOCK	287	782	23
MND/eGFP	327	856	12
MND/U 16RBE	161	736	8
	**(B) CD34+ Transduction efficiency**		
	% eGFP 1 day post TD		%eGFP 45 day post td
MND/eGFP	5.98		0.79
MND/U 16 RBE	3.24		0.42

The transduction efficiency (TD) was assessed by FACS analyses (monitoring eGFP expression) of the cells at days 1 and 45 post transduction (Table 1B). The TD was between 3 and 6 % of the gated population on day 1 post-transduction as determined by measuring eGFP expression. After 45 days, expression of eGFP dropped to less than 1%. The relatively low level of transduction efficiency is a consequence of the vector preparation and can vary significantly from experiment to experiment. The drop in eGFP expression is due to epigenetic silencing of the LTR driving EGFP in this vector. The Pol III promoter expression does not diminish during this time (H.Li, D. Castanotto and J. Rossi-unpublished observations).

Ten days after transduction the cells were sorted for eGFP expression. The sort purity was checked 5 days later with no significant differences among the various vector transduced cells (data not shown). Northern Blot analyses, carried out on total RNAs isolated from the eGFP sorted CD34+ derived cells, showed readily detectable levels of the U16RBE RNA, which were comparable to the levels of the endogenous U16 snoRNA (Fig. 4A).

The sorted cells were next challenged on the day of FACS analysis with the M-tropic HIV-1 JRFL isolate at a multiplicity of infection of 0.01. On days 1, 7, 14, 21 and 28 post-infection, the supernatants were collected and tested for p24^Gag ^protein accumulation.

As shown in figure 4B, while the mock and the eGFP sorted CD34+ cells transduced with the parental MND/EGFP vector were highly permissive to viral replication, cells expressing the MND/eGFP U16RBE resulted in over 4 logs of inhibition out to 28 days post infection.

## Discussion

We previously exploited the use of the U16 snoRNA as a vector for the nucleolar delivery of therapeutic anti-HIV ribozyme and TAR decoy RNAs [17,18]. It was previously demonstrated that substitution of the apical loop of U16 with the RBE sequence gave rise to an RNA decoy that localized in the nucleoli of 293 cells and was able to interact with Rev in *X. laevis *oocytes [14]. In these studies the interaction of the U16RBE decoy with Rev induced the re-localization of the decoy from the nucleus to the cytoplasm. Although we were not able to follow this trafficking in the present experiments, we assume this re-localization also took place in the T- cells and monocytes used in the present studies.

Our primary interest in the present studies was whether or not the U16RBE would sequester Rev sufficiently to inhibit HIV-1 replication in human CEM T-cells and in primary monocytes derived from hematopoietic progenitor CD34+ cells.

To test this possibility we inserted the U6 expression cassette containing the U16RBE sequence within the U3 region of the 3'LTR of two different retroviral vectors, pBabe puro and MND/eGFP (giving rise to pBabe/U16RBE and MND/eGFP U16RBE constructs, Fig. 1). Using the pBabe/U16RBE plasmid we first transduced the CEM T cell line and selected Puromycin resistant clones stably expressing the U16RBE (Fig. 2). We challenged some of these clones with HIV-1 _NL4-3 _and demonstrated that over a period of 17 days the decoy conferred near complete inhibition of viral replication (Fig. 3). To further test the U16RBE RNA as a possible candidate for hematopoietic stem cell gene therapy for HIV-1, the anti-HIV-1 activity of the U16RBE decoy was tested in human umbilical cord blood CD34+ derived monocytes. The CD34+ cells were transduced with either the MND/eGFP or MND/eGFP U16RBE constructs and allowed to differentiate into monocytes in culture. FACS sorted, transduced monocytes were challenged with HIV-1 _JRFL _at a multiplicity of infection of 0.01 and the infection was monitored for a period of 28 days (Fig. 4B). While the controls (mock and MND/eGFP transduced CD34+ cells) readily supported viral replication resulting in secretion of μg quantities of p24^Gag^, the CD34+ derived cells expressing the U16RBE showed four logs of p24 inhibition even after 28 days post infectious challenge (Fig. 4B).

In conclusion, we have demonstrated that the nucleolar localizing U16RBE decoy is a potent inhibitor of the HIV-1 replication. These results suggest that this inhibitor could be a candidate for anti-HIV gene therapy. The decoy could be used alone or in combination with other potent therapeutic RNAs, such as anti-HIV short hairpin RNAs [20]. Rev is essential for HIV-1 structural gene expression and full length viral RNA packaging and consequently sequestering of this protein via the decoy resulted in strong anti-HIV activity. It is highly likely that the decoy-Rev complex traffics to the cytoplasm as was observed in the *Xenopus *oocytes [14] since Crm1 is the transport carrier in both organisms. Targeting Rev in the nucleolus allows capturing of this protein prior to its interaction with the viral RRE. Furthermore, this strategy is unlikely to result in emergence of viral Rev mutants that can no longer bind the decoy since these would also be defective in binding to the viral RBE. In such a dual component system, mutants in Rev would have to co-evolve with altered forms of the RBE to escape the inhibitory activity of the decoy. Since the U16RBE is encoded in a small gene, it should be easy to combine this with other antiviral RNAs such as shRNAs. A combinatorial approach, which includes the U16RBE, should increase the overall antiviral potency [21] and further minimize the occurrence of viral escape mutants. Although the nucleolar localization of some HIV proteins [23], and perhaps of some classes of HIV RNAs [18], has been demonstrated, the functional role of this cellular localization is poorly understood. Concerning Rev, it has been suggested that the nucleoli could be a "storage site" for the protein [24] and in some cases it has been reported that Rev can function without nucleolar localization [25,26]. At the same time, it is noteworthy to mention that when Rev is expressed ectopically it localizes primarily within the nucleoli of human cells [2-4] where it multimerizes [10] and relocates CRM1 and some nucleoporins [9]. Rev multimerization is a critical event for the nucleocytoplasmic transport of incompletely spliced and unspliced HIV RNAs, and the association with CRM1 and nucleoporins is probably critical for its own nuclear export. These latter observations underline a crucial role of the nucleolus in Rev function and therefore in the HIV-1 replicative cycle. One possibility is that use of the Crm1 export pathway provides a mechanism for unspliced HIV RNAs to escape the nonsense mediated decay mechanism, although this is only a hypothetical explanation at this time. The importance of nucleolar trafficking of Rev and HIV is strengthened by the data reported here and by previously published data wherein we demonstrated that a nucleolar localizing TAR decoy [17] as well as a nucleolar localizing anti-HIV-1 hammerhead ribozyme [18] are both potent inhibitors of HIV-1 replication.

## References

[B1] Kingsman SM, Kingsman AJ (1996). The regulation of human immunodeficiency virus type-1 gene expression. Eur J Biochem.

[B2] Cullen BR, Hauber J, Campbell K, Sodroski JG, Haseltine WA, Rosen CA (1988). Subcellular localization of the human immunodeficiency virus trans-acting art gene product. J Virol.

[B3] Dundr M, Leno GH, Hammarskjold ML, Rekosh D, Helga-Maria C, Olson M (1995). The roles of nucleolar structure and function in the subcellular location of the HIV-1 Rev protein. J Cell Sci.

[B4] Stauber R, Gaitanaris GA, Pavlakis GN (1995). Analysis of trafficking of Rev and transdominant Rev proteins in living cells using green fluorescent protein fusions: transdominant Rev blocks the export of Rev from the nucleus to the cytoplasm. Virology.

[B5] Meyer BE, Malim MH (1994). The HIV-1 Rev trans-activator shuttles between the nucleus and the cytoplasm. Genes Dev.

[B6] Hope TJ (1999). The ins and outs of HIV Rev. Arch Biochem Biophys.

[B7] Fukuda M, Asano S, Nakamura T, Adachi M, Yoshida J, Yanagida M, Nishida E (1997). CRM1 is responsible for intracellular transport mediated by the nuclear export signal. Nature.

[B8] Fornerod M, Ohno M, Yoshida M, Mattaj IW (1997). CRM1 is an export receptor for leucine-rich nuclear export signals. Cell.

[B9] Zolotukhin AS, anf Felber BK (1999). Nucleoporins nup98 and nup214 participate in nuclear export of human immunodeficiency virus type 1 Rev. J Virol.

[B10] Daelemans D, Costes SV, Cho EH, Erwin-Cohen RA, Lockett S, Pavlakis GN (2004). In vivo HIV-1 Rev multimerization in the nucleolus and cytoplasm identified by fluorescence resonance energy transfer. J Biol Chem.

[B11] Bartel DP, Zapp ML, Green MR, Szostak JW (1991). HIV-1 Rev regulation involves recognition of non-Watson-Crick base pairs in viral RNA. Cell.

[B12] Heaphy S, Finch JT, Gait MJ, Karn J, Singh M (1991). Human immunodeficiency virus type 1 regulator of virion expression, rev, forms nucleoprotein filaments after binding to a purine-rich "bubble" located within the rev-responsive region of viral mRNAs. Proc Natl Acad Sci U S A.

[B13] Tiley LS, Malim MH, Tewary HK, Stockley PG, Cullen BR (1992). Identification of a high-affinity RNA-binding site for the human immunodeficiency virus type 1 Rev protein. Proc Natl Acad Sci U S A.

[B14] Buonuomo SB, Michienzi A, De Angelis FG, Bozzoni I (1999). The Rev protein is able to transport to the cytoplasm small nucleolar RNAs containing a Rev binding element. RNA.

[B15] Fragapane P, Prislei S, Michienzi A, Caffarelli E, Bozzoni I (1993). A novel small nucleolar RNA (U16) is encoded inside a ribosomal protein intron and originates by processing of the pre-mRNA. EMBO J.

[B16] Good PD, Krikos AJ, Li SXL, Bertrand E, Lee NS, Giver L, Ellington A, Zaia JA, Rossi JJ, Engleke DR (1997). Expression of small, therapeutic RNAs in human cell nuclei. Gene Ther.

[B17] Michienzi A, Li S, Zaia JA, Rossi JJ (2002). A nucleolar TAR decoy inhibitor of HIV-1 replication. Proc Natl Acad Sci U S A.

[B18] Michienzi A, Cagnon L, Bahner I, Rossi JJ (2000). Ribozyme-mediated inhibition of HIV 1 suggests nucleolar trafficking of HIV-1 RNA. Proc Natl Acad Sci U S A.

[B19] Kiss T (2002). Small nucleolar RNAs: an abundant group of noncoding RNAs with diverse cellular functions. Cell.

[B20] Hannon GJ, Rossi JJ (2004). Unlocking the potential of the human genome with RNA interference. Nature.

[B21] Li MJ, Bauer G, Michienzi A, Yee JK, Lee NS, Kim J, Li A, Castanotto D, Zaia JA, Rossi JJ (2003). Inhibition of HIV-1 infection by lentiviral vectors expressing Pol III-promoted anti-HIV RNAs. Mol Ther.

[B22] Morgestern JP, Land H (1990). Advanced mammalian gene transfer: high titre retroviral vectors with multiple drug selection markers and a complementary helper-free packaging cell line. Nucleic Acids Res.

[B23] Olson MOJ, Dundr M (2005). The moving parts of the nucleolus. Histochem Cell Biol.

[B24] Kjems J, Askjaer P (2000). Rev protein and its cellular partners. Adv Pharmacol.

[B25] McDonald D, Hope TJ, Parslow TG (1992). Posttranscriptional regulation by the human immunodeficiency virus type 1 Rev and human T-cell leukemia virus type I Rex proteins through a heterologous RNA binding site. J Virol.

[B26] Wolff H, Hadian K, Ziegler M, Weierich G, Kramer-Hammerle S, Kleinschmidt A, Erfle V, Brack-Werner R (2006). Analysis of the influence of subcellular localization of the HIV Rev protein on the Rev-dependent gene expression by multi-fluorescence live-cell imaging. Experimental Cell Research.

